# Cognitive offloading and metacognitive calibration in generative AI-mediated doctoral learning: a grounded theory study of accountable reconstruction

**DOI:** 10.3389/fpsyg.2026.1920947

**Published:** 2026-08-31

**Authors:** Lingyun Gao, Feng Zhang

**Affiliations:** School of Education, Beijing Institute of Technology, Beijing, China

**Keywords:** accountable reconstruction, cognitive offloading, doctoral learning, generative artificial intelligence, metacognitive calibration

## Abstract

**Background:**

Generative artificial intelligence (GenAI) is increasingly used to explain concepts, summarize literature, suggest methods, generate arguments, and revise academic writing. These functions may reduce entry barriers to complex learning tasks while reshaping the cognitive and metacognitive work through which learners develop understanding. Doctoral research learning provides a high-complexity context for examining this issue because students must move from understanding existing knowledge to producing, justifying, and defending original claims.

**Objective:**

This study examines how GenAI-mediated cognitive offloading redistributes cognitive work in doctoral research learning, how it may support or weaken learner-controlled engagement, and how learners recalibrate and reconstruct AI-supported outputs into defensible understanding and research judgment.

**Methods:**

A grounded theory design was adopted. After four pilot interviews were used to refine the interview protocol, formal data were collected from 28 semi-structured interviews, 42 voluntarily provided AI-use artifacts, and 12 stimulated-recall discussions. Open coding, axial coding, selective coding, constant comparison, theoretical sampling, and theoretical saturation testing were used to construct a mechanism model. Interview transcripts were coded in Chinese to preserve participants' original meanings, and representative quotations were translated into English after analysis.

**Results:**

The analysis generated a five-part mechanism consisting of task-entry scaffolding, modular generation, metacognitive calibration crisis, critical reconstruction, and research accountability. Episodes reflected more learner-controlled engagement when GenAI reduced entry difficulty while preserving students' reading, verification, and reconstruction. Substitution risk appeared when AI-generated frameworks, method suggestions, thesis structures, or texts appeared coherent before learners had developed corresponding understanding. Calibration crisis appeared when recognition was mistaken for understanding, fluency for mastery, and coherence for justification. Critical reconstruction addressed this risk through source verification, self-explanation, contextual adaptation, and counterargument generation.

**Conclusion:**

Learner-controlled engagement was most evident in episodes where doctoral students transformed AI-generated outputs into owned understanding, calibrated judgment, and defensible research decisions. These episodes suggest that accountable reconstruction helps preserve learners' responsibility for verification, reasoning, and final judgment in GenAI-supported doctoral learning.

## 1 Introduction

Generative artificial intelligence (GenAI) has rapidly become embedded in higher education learning and academic work. GenAI tools can explain concepts, summarize literature, support writing, generate examples, compare ideas, produce feedback, and assist research planning. Early studies of conversational GenAI in education have shown that learners and educators recognize its potential for personalized support, academic writing assistance, brainstorming, and research-related tasks, while also expressing concerns about reliability, ethical use, overreliance, academic integrity, and assessment validity (Lo, 2023; Tlili et al., 2023; Chan and Hu, 2023; Kasneci et al., 2023). More recent work has shifted attention from initial tool adoption to questions concerning responsible use, student agency, doctoral writing, supervisory practice, and the quality of learning processes in GenAI-mediated academic work (Darvishi et al., 2024; Fan et al., 2025; Akbar, 2025; McKenna, 2025).

The educational implications of GenAI are shaped by how learners use it within cognitive and metacognitive activity. GenAI systems generate explanations, organize conceptual relations, propose methodological options, produce arguments, and polish academic text. These capabilities create different forms of learning support. A learner may use GenAI to enter a difficult knowledge domain and then deepen understanding through reading, verification, and reconstruction. Another learner may accept a coherent AI-generated output as if it already represented their own understanding. The same tool can therefore support learner-controlled engagement or reduce the learner's involvement in the cognitive and metacognitive work through which understanding is formed.

This ambiguity is closely related to established concerns in learning science. Cognitive load theory suggests that instructional support can be beneficial when it reduces unnecessary processing and allows learners to focus on meaningful learning activities (Sweller et al., 2011). Cognitive offloading research has shown that external tools can redistribute cognitive work, and the educational meaning of such redistribution depends on the kind of mental work that is offloaded and the quality of learners' monitoring (Risko and Gilbert, 2016; Dunn and Risko, 2016). Self-regulated learning research similarly emphasizes that effective learning depends on learners' ability to monitor understanding, evaluate progress, and adjust strategies (Zimmerman, 2002). In GenAI-supported learning, a central psychological issue is how learners monitor the gap between generated fluency and owned understanding. Monitoring this gap is important because learners' judgments of their own understanding guide subsequent regulation, verification, and strategy adjustment. When AI-generated outputs appear fluent, plausible, and complete, learners may overestimate their comprehension and reduce the reconstruction work needed for durable understanding and defensible judgment (Fan et al., 2025; Xu et al., 2025).

AI literacy has also become an important concept for understanding learning with intelligent systems. AI literacy includes the ability to understand AI systems, evaluate outputs, recognize limitations, and use AI tools responsibly (Long and Magerko, 2020; Ng et al., 2021). In research-intensive learning contexts, AI literacy also involves the ability to decide whether AI-generated outputs can be transformed into defensible knowledge claims. Learners must judge, verify, revise, and take responsibility for the intellectual work that AI helps them produce.

Doctoral research learning represents a high-complexity form of academic learning because students must move from understanding existing knowledge to producing, justifying, and defending original claims. Doctoral students synthesize literature, formulate research questions, justify theoretical choices, select methods, interpret evidence, construct arguments, and respond to supervisory and disciplinary scrutiny. Their learning is therefore inseparable from the formation of research judgment. Recent discussions of doctoral education in the age of GenAI have emphasized that doctoral development concerns knowledge products and the formation of the doctoral knower (McKenna, 2025). Recent studies of doctoral writing and dissertation practices further show that GenAI is increasingly used in meaning negotiation, thesis writing, authorial stance construction, and dissertation-related language support (Parker et al., 2025; Hoomanfard and Shamsi, 2025; Zhao, 2025). Related evidence from school-based science learning also shows that students' engagement with GenAI may move from initial trust toward critical reconstruction of AI-generated content (Zhou, 2026). This makes doctoral research learning a particularly important context for examining how AI-supported outputs are transformed into accountable understanding and defensible research decisions.

In this context, GenAI can generate outputs that resemble the external form of research expertise before students have developed the judgment required to own that expertise. It can produce a literature map before the student has read the literature, a theoretical framework before the student has understood the conceptual relations, a method recommendation before the student has examined assumptions and data fit, and a fluent paragraph before the student can defend the claim expressed in it. Even plausible or useful AI output may become risky when doctoral students mistake generated coherence for their own research understanding.

Existing GenAI research has examined learning support, student experience, writing assistance, academic integrity, responsible use, and institutional policy in higher education (Cotton et al., 2024; Farrokhnia et al., 2024). Recent empirical work has also begun to examine how GenAI affects metacognitive laziness, self-regulated learning, cognitive offloading, and doctoral students' AI-supported research practices (Fan et al., 2025; Akbar, 2025; Xu et al., 2025). These studies provide an important basis for understanding the educational uptake of GenAI. Yet less is known about how GenAI-mediated cognitive offloading shapes metacognitive calibration and self-regulated reconstruction in high-complexity doctoral research learning. In particular, more attention is needed to how AI-generated fluency and coherence affect learners' judgment of their own understanding, and how learners transform AI-supported outputs into knowledge claims, methods, and arguments they can explain, verify, and defend.

This study addresses that gap through a grounded theory investigation of GenAI-mediated doctoral learning. It focuses on the process through which doctoral students transform AI-supported outputs into research decisions they can justify, defend, and own. The central concern is how the redistribution of cognitive work between doctoral students and GenAI relates to learner ownership, metacognitive monitoring, and reconstruction in complex research tasks.

The study is guided by three research questions:

*RQ1:* How do doctoral students use GenAI to support high-complexity research learning tasks?
*RQ2:* How does the redistribution of cognitive work between doctoral students and GenAI shape their engagement with complex research tasks?
*RQ3:* How do learners recalibrate understanding and reconstruct AI-supported outputs into defensible research judgment?

In this study, GenAI-mediated cognitive offloading refers to the redistribution of conceptual, methodological, or textual work from the learner to AI-supported external resources. This definition builds on cognitive offloading research, which conceptualizes external tools as resources that redistribute cognitive work beyond internal memory and reasoning (Risko and Gilbert, 2016; Dunn and Risko, 2016). Cognitive substitution refers to a situation in which such redistribution replaces the learner's own reasoning rather than supporting it. The manuscript uses cognitive offloading as the core theoretical term. Although adjacent work has used the language of “outsourcing” cognitive tasks to technologies or other agents when discussing cognitive offloading and intellectual agency (Turner, 2022), the present manuscript uses cognitive outsourcing only as a descriptive umbrella expression for delegating task responsibility to an external agent. It is not treated as a formal coding category or used interchangeably with cognitive offloading. Metacognitive calibration crisis refers to learners' misjudgment of the gap between AI-generated coherence and their own understanding, drawing on self-regulated learning research on monitoring, evaluation, and strategy adjustment (Zimmerman, 2002) and recent GenAI research on metacognitive laziness and metacognitive support (Fan et al., 2025; Xu et al., 2025). Critical reconstruction refers to self-regulated practices through which learners verify, adapt, explain, and challenge AI-supported outputs. This concept is informed by self-regulated learning and AI literacy research, while its specific components emerged from the empirical analysis (Long and Magerko, 2020; Ng et al., 2021). In this study, research accountability refers to the extent to which learners can defend final claims, methods, and arguments as their own scholarly judgment. Accountable reconstruction is the study-derived core category that describes how doctoral students transform AI-supported outputs into owned understanding, calibrated judgment, and defensible research decisions. The analysis focuses on process-level indicators of learner-controlled engagement, including learners' capacity to monitor AI-generated outputs, reconstruct the underlying reasoning, and justify the resulting claims, methods, or arguments. Doctoral research agency is understood as the capacity to transform external support into accountable research decisions within disciplinary and supervisory contexts (McKenna, 2025; Parker et al., 2025).

## 2 Materials and methods

### 2.1 Research design

This study adopted a grounded theory approach because it aimed to construct an explanatory model of an emerging educational process. Grounded theory was appropriate because the study sought to explain process-level learning mechanisms grounded in learners' concrete AI-mediated research episodes. GenAI-mediated doctoral learning is a rapidly developing practice for which existing theoretical categories remain incomplete. The study therefore focused on process and mechanism, with close attention to how students interpreted, used, revised, and justified AI-generated outputs in concrete research tasks.

The study primarily followed a procedural grounded theory logic while drawing on broader quality criteria for credibility, analytic fit, and theoretical usefulness (Corbin and Strauss, 1990; Chun Tie et al., 2019; Charmaz and Thornberg, 2021). Cognitive offloading, metacognitive calibration, and self-regulated learning were used as sensitizing ideas rather than as *a priori* coding categories. These concepts informed the formulation of the research questions and oriented attention to possible learning mechanisms, but the coding structure was developed through open coding, constant comparison, theoretical sampling, and repeated comparison of empirical incidents, artifact episodes, analytic memos, and emerging category relations. The categories reported in the Results section were generated through comparison across interview accounts, AI-use artifacts, stimulated-recall explanations, negative cases, and saturation-test interviews.

The study combined three sources of data: semi-structured interviews, AI-use artifacts, and stimulated-recall discussions. AI-use artifacts included prompt-response excerpts, AI-generated outlines, AI-generated conceptual frameworks, method-suggestion records, revised writing traces, and AI-assisted coding or data-analysis examples voluntarily provided by participants. Stimulated recall was used to ask participants to explain why they entered particular prompts, how they interpreted AI responses, which parts they accepted or rejected, and how they verified or revised AI-generated outputs. This design allowed the analysis to examine concrete episodes of AI-mediated learning together with participants' interpretations of those episodes.

### 2.2 Participants and data collection

A total of 32 doctoral students were interviewed. Four doctoral students first participated in pilot interviews, which were used to refine the interview guide and adjust the questioning sequence. These pilot interviews were excluded from formal coding and analysis. The formal analytic sample consisted of 28 doctoral students from six Chinese research universities.

Participants were recruited through purposive maximum-variation sampling, theoretical sampling, and limited snowball sampling. Initial participants were accessed through graduate-level academic networks, doctoral-student groups, supervisor referrals, and peer recommendations across six research universities. Recruitment messages briefly introduced the purpose of the study, inclusion criteria, voluntary participation, confidentiality protection, and the option to share AI-use artifacts. To support maximum variation, recruitment sought diversity in disciplinary field, doctoral year, gender, university context, and reported GenAI-use experience. As analysis progressed, later recruitment and follow-up questioning placed greater emphasis on participants who reported framework generation, method suggestion, AI-supported writing, or uncertainty about AI-generated outputs. These cases helped elaborate the emerging distinction between scaffolding and substitution.

The 28 formal semi-structured interviews were conducted between March and June 2026. Each interview lasted between 48 and 72 min, with a mean duration of 59.8 min and a standard deviation of 6.9 min. Interviews were conducted in Chinese, audio-recorded with consent, and transcribed verbatim. The interview guide covered seven areas: doctoral research background, history of GenAI use, GenAI-supported research tasks, perceived support and risks, verification and reconstruction practices, supervisory or institutional guidance, and tool/model context. Participants were first invited to provide open-ended accounts of specific GenAI-supported research episodes in their own terms. The interview guide was applied flexibly rather than as a fixed sequence, and the construct-oriented questions were used only as optional follow-up probes to clarify or elaborate participants' accounts. These prompts guided data elicitation but were not used as an *a priori* coding framework. The participant characteristics, data sources, and analytic roles of interviews are summarized in Table 1.

**Table 1:** Participant and data characteristics.

Characteristic	Category	*n*
Interview stage	Pilot interviews excluded from formal analysis	4
Interview stage	Formal interviews included in analysis	28
Interview stage	Total doctoral students interviewed	32
Gender of formal participants	Female	14
Gender of formal participants	Male	14
Doctoral year	Year 1	5
Doctoral year	Year 2	10
Doctoral year	Year 3	8
Doctoral year	Year 4	5
Disciplinary field	Humanities and social sciences	15
Disciplinary field	STEM and applied sciences	10
Disciplinary field	Interdisciplinary fields	3
Additional data	Participants providing AI-use artifacts	12
Additional data	AI-use artifacts collected	42
Additional data	Stimulated-recall discussions	12
Analytic role	Model construction interviews	22
Analytic role	Theoretical saturation test interviews	6

To contextualize participants' AI-mediated learning episodes, the study recorded both GenAI tools and visible model/version labels. In this study, “tool” refers to the user-facing product, platform, or application through which participants interacted with GenAI, whereas “model/version” refers to the model label, product-family label, or application-version information reported by participants or visible in AI-use artifacts. At the aggregate level, the most frequently reported tools were ChatGPT (*n* = 23), DeepSeek (*n* = 14), Google Gemini (*n* = 9), Wenxin Yiyan/ERNIE Bot (*n* = 6), Claude (*n* = 5), Doubao (*n* = 4), and GitHub Copilot (*n* = 3). Because several participants used more than one tool across different research tasks, tool use was recorded as multiple-response information. The model/version labels represented in the data included ChatGPT with GPT-5, DeepSeek-R1, DeepSeek-V3, Gemini 2.5 Pro, Gemini 2.5 Flash, Claude Sonnet 4.5, ERNIE 4.5, Doubao-1.5-Pro, and GitHub Copilot Chat in IDE extension/plugin environments. GitHub Copilot was treated as an AI coding assistant rather than as a general-purpose conversational chatbot. Participant-level tool and model/version profiles are provided in Supplementary Table S1, and the interview and stimulated-recall prompts used to elicit tool/model context are provided in Supplementary Table S2. These data were used to contextualize AI-mediated learning episodes rather than to compare model-level performance.

In addition, 42 AI-use artifacts were voluntarily provided by 12 participants. Follow-up stimulated-recall discussions were conducted with these 12 participants because their artifacts enabled episode-level reconstruction of concrete AI-supported research activities. These participants were not treated as a separate subsample for prevalence estimation. They were selected for stimulated recall because they provided usable artifacts and represented variation in task type, discipline, doctoral year, and AI-use pattern. The stimulated-recall discussions lasted between 20 and 36 min, with a mean duration of 27.9 min and a standard deviation of 4.8 min.

In this study, an artifact-based episode refers to a bounded AI-mediated research event that could be linked to a participant's prompt purpose, AI-generated output, participant annotation or retrospective explanation, and subsequent decision to accept, reject, revise, verify, or adapt the output. Each artifact-based episode was therefore treated as process evidence showing how a specific AI output entered the learner's research practice. During stimulated recall, participants were invited to annotate or explain specific AI-generated outputs, including parts they accepted, questioned, ignored, revised, or later found problematic. Artifact-based episodes deepened mechanism-level interpretation and clarified how AI outputs entered participants' research learning practices. They were used to support process interpretation rather than to estimate the prevalence of specific AI-use behaviors across the full sample.

Interview transcripts were coded in Chinese to preserve participants' original meanings. Representative quotations were translated into English after analysis by the authors and then checked against the original Chinese transcripts for semantic accuracy. Minor edits were made to improve readability while preserving substantive meaning. Representative quotations are labeled by participant identifier and data source. Quotations from formal semi-structured interviews are marked as Pxx[Interview], whereas quotations from stimulated-recall discussions linked to AI-use artifacts are marked as Pxx[Artifact].

### 2.3 Data analysis

The analysis proceeded through open coding, axial coding, selective coding, constant comparison, and theoretical saturation testing.

During open coding, 512 meaning units were identified from the 22 model-construction interviews and related artifacts. These meaning units were condensed into 63 initial concepts through line-by-line and incident-by-incident comparison. Participant language and incident-level descriptions were coded before comparison with sensitizing concepts. Theoretical labels were finalized after repeated comparison among interview incidents, artifact episodes, analytic memos, and emerging category relations. This process supported the empirical grounding of the categories.

Initial concepts were then grouped into 19 initial categories. Coding focused on the location of cognitive work. The analysis examined which parts of the task were supported by AI, which parts were retained by the learner, and how learners monitored and addressed the gap between generated output and owned understanding.

During axial coding, the 19 initial categories were compared in terms of their conditions, actions, and consequences in GenAI-mediated doctoral learning. This process generated five main categories: task-entry scaffolding, modular generation, metacognitive calibration crisis, critical reconstruction, and research accountability. These categories captured recurrent relations among AI-supported task entry, AI-generated research components, miscalibration, reconstruction, and accountability.

During selective coding, the five main categories were integrated around the core category: accountable reconstruction in GenAI-mediated doctoral learning. The emerging model explained how cognitive offloading could become productive scaffolding, how it could shift into cognitive substitution, and how students re-established ownership through critical reconstruction. The coding development and audit trail are summarized in Supplementary Table S3.

The 12 stimulated-recall discussions were integrated into the coding process at the episode level. For participants who provided artifacts, interview segments, artifact traces, participant annotations, and stimulated-recall explanations were linked by participant and task and coded together to examine how AI-generated outputs were interpreted, accepted, revised, rejected, verified, or reconstructed. Stimulated recall was therefore used to refine category properties and clarify process relations rather than to create a separate coding scheme independent from interview data.

Two researchers reviewed the coding structure. Before discussion, raw percentage agreement for main-category assignment across 120 double-coded meaning units was 0.86, following practical guidance on transparency in qualitative coding review (O'Connor and Joffe, 2020). Disagreements were resolved through comparison with the original transcript segments, artifact episodes, and analytic memos. The agreement value was used as a transparency indicator rather than as a substitute for interpretive agreement in the grounded theory analysis.

**Figure 1:**
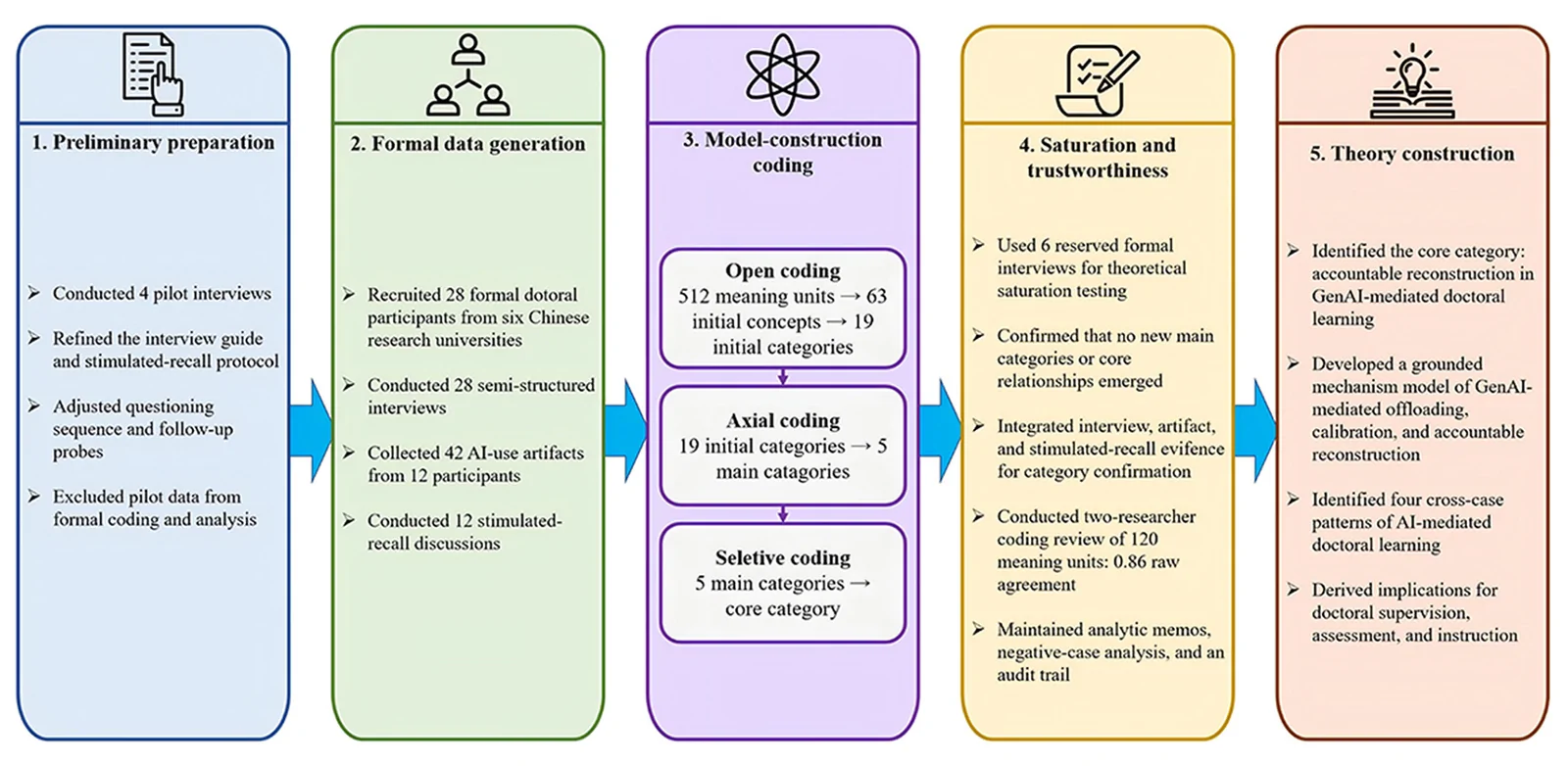
Research procedure and grounded-theory analysis of GenAI-mediated doctoral learning. The figure summarizes the analytic procedure. Although displayed sequentially for clarity, coding, constant comparison, memoing, negative-case analysis, and category refinement were iterative throughout the analysis. Pilot interviews were used only to refine the interview guide and stimulated-recall protocol and were excluded from formal coding and analysis.

The six reserved formal interviews were selected before saturation testing to preserve variation across discipline, doctoral year, AI-use task type, and artifact availability. They included two participants from humanities and social sciences, two from STEM and applied sciences, and two from interdisciplinary fields, and included both artifact and non-artifact cases. These interviews were not used to construct the initial model, but to test whether the five main categories, their properties, and their relationships were sufficiently developed. No new main categories or core relationships emerged from these six interviews (Hennink et al., 2017; Saunders et al., 2018). Trustworthiness strategies and saturation checks are summarized in Supplementary Table S4, and the overall research procedure is shown in Figure 1.

### 2.4 Trustworthiness and ethics

Several strategies were used to enhance trustworthiness. First, data triangulation was achieved by combining interviews, artifacts, and stimulated recall. Second, constant comparison was used across participants, disciplines, doctoral stages, task types and AI-use patterns. Third, negative cases were examined, especially participants who used AI frequently while maintaining strong agency and participants who used AI less frequently while showing limited conceptual or methodological ownership. These comparisons clarified that differences in learner ownership were linked to reconstruction practices rather than to AI-use frequency alone. Fourth, analytic memos were maintained throughout coding to record category development and theoretical decisions. Fifth, preliminary interpretations were discussed by the research team to refine category boundaries. Sixth, the translation of representative quotations was checked against the original Chinese transcripts to preserve analytic meaning across languages.

Formal member checking of the final categories was not conducted. Several participants discussed unpublished doctoral research ideas, draft arguments, method choices, or AI-use records that they considered sensitive. Returning the final category structure to participants could also have shifted participants from explaining their own AI-use episodes to evaluating the theoretical adequacy of the emerging model. To protect confidentiality and maintain analytic independence, the study used stimulated recall, artifact-based comparison, negative-case analysis, two-researcher coding review, and analytic memoing to strengthen interpretive credibility.

The study received ethical approval from the authors' institution. The study was conducted in accordance with local legislation and institutional requirements. Participants provided written informed consent and were informed that participation was voluntary and that they could withdraw at any time. Interview transcripts and AI-use artifacts were anonymized. No original AI-use artifact containing identifiable research data, unpublished results, or personal information is reproduced in the manuscript. Participant identifiers are reported as P01 to P28.

## 3 Results

### 3.1 From empirical materials to category structure

The grounded theory analysis generated five main categories from 19 initial categories. Table 2 presents the relationship between the main categories, initial categories, and analytical focus. It shows how the empirical categories were organized around the process through which GenAI-mediated cognitive offloading became task-entry scaffolding, modular generation, metacognitive calibration crisis, critical reconstruction, and research accountability. Representative empirical materials and initial concepts for all 19 initial categories are provided in Supplementary Table S5.

**Table 2:** Main categories and analytical focus.

Main category	Initial categories	Analytical focus
B1 Task-entry scaffolding	Literature orientation; concept clarification; cross-disciplinary translation; writing-entry support	How GenAI reduces entry difficulty while preserving learner-controlled cognitive engagement.
B2 Modular generation	Framework generation; method suggestion; thesis structure production; text production	How AI-generated coherence may precede owned understanding.
B3 Metacognitive calibration crisis	Premature coherence; illusion of understanding; overconfidence in fluency; blurred conceptual boundaries	How generated fluency creates metacognitive miscalibration.
B4 Critical reconstruction	Source verification; self-explanation; contextual adaptation; counterargument generation	How learners use self-regulated strategies to transform AI output into owned understanding.
B5 Research accountability	Epistemic responsibility; methodological ownership; supervisory accountability	How reconstructed AI-supported outputs become claims, methods, or arguments that learners can justify and defend.

These categories describe recurrent relations among AI-supported task entry, AI-generated research modules, possible miscalibration, and practices of reconstruction and accountability. The educational meaning of GenAI depended on where cognitive work was located. AI could reduce entry difficulty while preserving student judgment, and it could also relocate intellectual labor away from the learner. The categories should be understood as analytically related processes rather than as a fixed sequence that every participant followed in the same order. In some episodes, task-entry scaffolding led to deeper verification. In others, modular generation created premature coherence and required later reconstruction. Some participants moved back and forth between scaffolding, generation, checking, and accountability within the same task.

### 3.2 Task-entry scaffolding: productive offloading for entering research uncertainty

Task-entry scaffolding describes how GenAI helped doctoral students enter difficult research tasks. Participants used AI to map unfamiliar literature, clarify concepts, translate ideas across disciplinary boundaries, and reduce initial writing difficulty. In this use pattern, cognitive offloading lowered entry barriers while preserving the learner's own research judgment.

P03, a first-year sociology doctoral student, described AI as a reading map:

“*Before reading a dense theoretical article, I ask a GenAI tool to outline the main debate and explain the key concepts. I do not use the answer as a substitute for the article. It is more like a map. After I get the map, I know where to pay attention when I read the original text*.” (P03[Interview])

The artifact provided by P03 showed a prompt asking AI to summarize a theoretical debate and identify key conceptual tensions. In the stimulated-recall discussion, P03 explained that she used the response to mark sections of the original article for close reading. This episode shows AI functioning as an entry scaffold because the output helped the participant approach the original text more strategically while keeping the original text as the primary object of learning.

Task-entry scaffolding also appeared in cross-disciplinary work. P10, a materials science doctoral student, explained:

“*My project involves materials chemistry and mechanical behavior. Sometimes I understand the experiment but not the theoretical explanation from another field. I ask AI to explain the mechanism in simpler language or compare two possible explanations. It helps me cross the language boundary between fields*.” (P10[Interview])

P10′s artifact included an AI-generated comparison between two mechanisms explaining material fatigue behavior. In stimulated recall, he marked several phrases as “too general” and explained how he checked them against recent domain papers. AI supported interdisciplinary translation, while field-specific verification remained central.

Writing-entry support was another common form of scaffolding. P07 noted:

“*I usually write the paragraph myself first. Then I ask AI to make the expression clearer. If I let AI write first, the paragraph becomes too general. If I write first and then use AI, it helps me express my own meaning better*.” (P07[Interview])

These cases show how doctoral students used AI to orient attention, clarify terminology, compare possible explanations, and polish self-generated writing. Task-entry scaffolding therefore represents productive cognitive offloading, because GenAI reduced extraneous entry difficulty while preserving learner-controlled reading, verification, and reconstruction. This pattern appeared across literature orientation, concept clarification, cross-disciplinary translation, and writing-entry support, with discipline-specific variation in the form of scaffolding.

### 3.3 Modular generation: generated coherence before owned understanding

Modular generation describes how GenAI directly produced visible components of doctoral research work. Participants reported using AI to generate conceptual frameworks, method suggestions, thesis structures, hypotheses, chapter outlines, and polished paragraphs. These outputs often looked coherent and professional, and their implications for learner engagement depended on how students examined and reconstructed the reasoning embedded in them.

P05, a management doctoral student, described this experience:

“*AI can generate a theoretical framework in seconds. It gives variables, relationships, and even possible hypotheses. When I first see it, I feel the framework is complete. But when my supervisor asks why these variables should be connected, I realize I cannot explain the logic. I only know that the structure looks reasonable*.” (P05[Artifact])

A closer artifact-based episode illustrates this process. P05 asked GenAI to construct a theoretical framework for an early-stage research idea. The prompt asked the system to identify possible variables, relationships, and hypotheses. The AI output presented a set of linked constructs and hypothesis-like statements, including suggested directional relationships among institutional conditions, individual perceptions, and research outcomes. In the artifact, P05 later marked several links as “unclear source” and “needs literature support.” During stimulated recall, she explained that the output “looked like a model” but that she could not explain why some variables should be connected. Additional de-identified artifact episodes are provided in Supplementary Table S6.

The episode shows how AI may generate the external form of theory before the student has developed the reasoning needed to defend it. The framework became risky when its coherence was mistaken for theoretical justification.

Method suggestion created a similar risk. P12 explained:

“*When I ask AI what model I should use, it gives several choices and explains them confidently. But it does not know my data problems. Sometimes the method sounds advanced but does not fit the data*.” (P12[Artifact])

P12's artifact showed AI recommending several statistical models. Some suggestions were plausible in general but inappropriate for his data structure. The AI output expanded possible options, while contextual methodological judgment remained with the learner.

P09 reported a related experience with dissertation structure:

“*I asked AI to reorganize my chapter structure. It gave me a very neat outline. The problem was that the outline made my thesis look smoother than it actually was. It covered the gaps. Later I found some transitions were not real transitions. They were just nice headings*.” (P09[Artifact])

Modular generation involves AI-generated research components that may enter the core of doctoral knowledge production. It is educationally consequential because it changes the learner's perception of task completion. The output may look complete before the learner has completed the conceptual, methodological, or argumentative work required to own it. This category marks the point at which cognitive offloading may shift from supporting task entry to replacing parts of the intellectual work through which research judgment is formed. This pattern appeared across framework generation, method consultation, dissertation structuring, and text production, which suggests that generated coherence may occur in conceptual, methodological, organizational, and linguistic forms of research work.

### 3.4 Metacognitive calibration crisis: when generated coherence exceeds owned understanding

Metacognitive calibration crisis captures the gap between the apparent coherence of AI outputs and students' actual understanding. Participants repeatedly described situations in which AI-generated texts, frameworks, or explanations appeared fluent and complete, leading them to overestimate their own comprehension or research readiness.

P08, a philosophy doctoral student, described this clearly:

“*The paragraph was so fluent that I thought I understood the concept. But later I found I could only repeat the AI's wording. I could not reconstruct the argument in my own language. In philosophy, that means I did not really understand it*.” (P08[Interview])

This episode shows that recognizing an AI explanation as plausible differs from being able to reconstruct the reasoning behind it. The difference between recognition and reconstruction was a central feature of calibration crisis.

P06, a psychology doctoral student, emphasized conceptual boundary problems:

“*AI can explain constructs very clearly. But when I compare the explanation with original scale papers, I find that it sometimes mixes related concepts. The explanation sounds clear, but the boundary is not precise. If I do not check, I may think I understand the construct*.” (P06[Artifact])

The artifact provided by P06 showed AI explaining two related psychological constructs in a simplified way. The participant highlighted parts where AI blurred distinctions between construct definition, measurement indicators, and theoretical antecedents. Calibration crisis therefore appeared in factual, conceptual, and methodological forms.

The illusion of understanding was also connected to emotional relief. P01, an education doctoral student, explained:

“*When I feel anxious because there are too many papers, AI gives me a sense of control. It summarizes quickly. I feel I have covered the field. But later I realize I only covered AI's version of the field*.” (P01[Interview])

This account shows how GenAI can regulate anxiety and create a sense of progress. The feeling of control became miscalibrated when it was based mainly on AI-generated summaries rather than engagement with the literature itself.

Across cases, calibration crisis appeared when recognition was mistaken for understanding, fluency for mastery, and coherence for justification. For doctoral students, this crisis is especially consequential because the object of miscalibration is often a research claim, method, or argument that must later be defended. When left unaddressed, calibration crisis signaled substitution risk, characterized by surface productivity with limited conceptual ownership or methodological justification. This category appeared across both early-stage and more advanced doctoral cases, although early-stage participants more often described conceptual miscalibration, whereas advanced participants more often described methodological or argumentative miscalibration.

### 3.5 Critical reconstruction: transforming AI output into owned understanding

Critical reconstruction describes the practices through which doctoral students redirected AI-supported work toward accountable learning. Critical reconstruction included source verification, self-explanation, contextual adaptation, and counterargument generation. These practices helped students transform AI-generated outputs into materials they could evaluate, revise, and own.

Verification was the most common practice. P07 explained:

“*When AI gives me a statement about language learning, I ask where this idea comes from. If it cannot give reliable sources, I search myself. I do not cite AI. I cite the paper after I read it*.” (P07[Interview])

In this case, AI became a prompt for further source checking. Several participants described similar practices: asking AI for possible terms, using those terms to search databases, comparing AI claims with original papers, and discarding AI-generated claims that could not be verified.

P16, an environmental science doctoral student, made a similar point about methods:

“*AI may suggest a method, but I check whether recent papers in my field actually use it. I also check whether the assumptions fit my data. AI is like a starting point, not evidence*.” (P16[Artifact])

This excerpt highlights the difference between option generation and methodological judgment. AI can generate possible methods, while the learner must examine data conditions, assumptions, disciplinary expectations, and research design.

Self-explanation was another important strategy. P03 described a practice of closing the AI page and trying to explain the concept independently:

“*If AI explains a theory, I close the page and try to explain it myself. If I cannot explain it without AI, I know I have not understood it. Then I go back to the original text*.” (P03[Interview])

Self-explanation functioned as a metacognitive test. It helped students distinguish between familiarity with AI wording and genuine understanding.

Some participants used AI to generate counterarguments. P11 said:

“*I ask AI to criticize my argument. Then I ask it to defend the opposite position. This helps me see weak points. But I do not copy the answer. I use it to revise my own argument*.” (P11[Artifact])

In this use pattern, AI became a dialogic tool for testing thought. Critical reconstruction involved transforming AI-generated material into something the learner could evaluate, adapt, explain, and defend.

Critical reconstruction therefore represents a self-regulated learning process in which learners monitor AI-generated outputs, control their use, and transform them into owned understanding. Source verification functioned as epistemic monitoring, self-explanation as calibration checking, contextual adaptation as strategic control, and counterargument generation as reflective evaluation. This category functioned as the process through which participants addressed calibration crisis and substitution risk. These reconstruction practices appeared across humanities and social sciences, STEM, and interdisciplinary cases, although their specific forms differed by task type. Humanities and social sciences participants more often emphasized conceptual explanation and source verification, whereas STEM and applied-science participants more often emphasized methodological fit, data conditions, and technical assumptions.

### 3.6 Research accountability: from reconstructed understanding to defensible judgment

Research accountability describes how doctoral students assumed responsibility for final research claims, methods, and arguments. Research accountability was the point at which AI-supported learning became visible as owned understanding and defensible judgment. Whereas critical reconstruction refers to the practices through which students work on AI-generated outputs, research accountability refers to the point at which reconstructed outputs become claims, methods, or arguments that students are prepared to defend. In this sense, research accountability is a process-level indicator of learner ownership and accountable engagement in high-complexity research learning. It indicates learners' metacognitive awareness of what they understand, what remains uncertain, and what they are able to defend.

Epistemic responsibility was most visible when participants distinguished between AI-generated possibilities and claims they were willing to include in their own research. P18 explained:

“*AI can give me possible directions, but I cannot put a claim into my paper unless I have read the original source and can explain why it supports my argument*.” (P18[Artifact])

In this sense, AI-generated statements became usable after they were reconnected to literature, evidence, and the participant's own argumentative purpose. AI could suggest possible claims, while responsibility for turning those suggestions into defensible research statements remained with the student.

Methodological ownership appeared in a related way. P02, a doctoral student in an empirical science field, explained:

“*I ask AI for potential methods, but I decide whether they fit my equipment, data, and research question. AI gives options. It does not know my lab conditions. If the method is wrong, I cannot say AI suggested it. It is my responsibility*.” (P02[Interview])

This excerpt illustrates methodological ownership. AI may generate options, while the doctoral student evaluates assumptions, contextual fit, and disciplinary legitimacy.

Participants also described supervisory accountability as an important condition under which AI-supported work became more visible and open to justification. P14 reported:

“*My supervisor does not simply say AI is forbidden. He asks me to mark which parts were influenced by AI and explain why I accepted or rejected the suggestions. This makes me more careful. I have to understand the suggestion before using it*.” (P14[Artifact])

In participants' accounts, supervisory questioning made AI use visible as a learning and research process. It required students to explain the relation between AI outputs, source evidence, methodological decisions, and final arguments. Research accountability was most visible in episodes where students had to reconnect AI-supported outputs to source evidence, data conditions, or supervisory questioning.

### 3.7 Mechanism model of GenAI-mediated offloading, calibration, and accountable reconstruction

**Figure 2:**
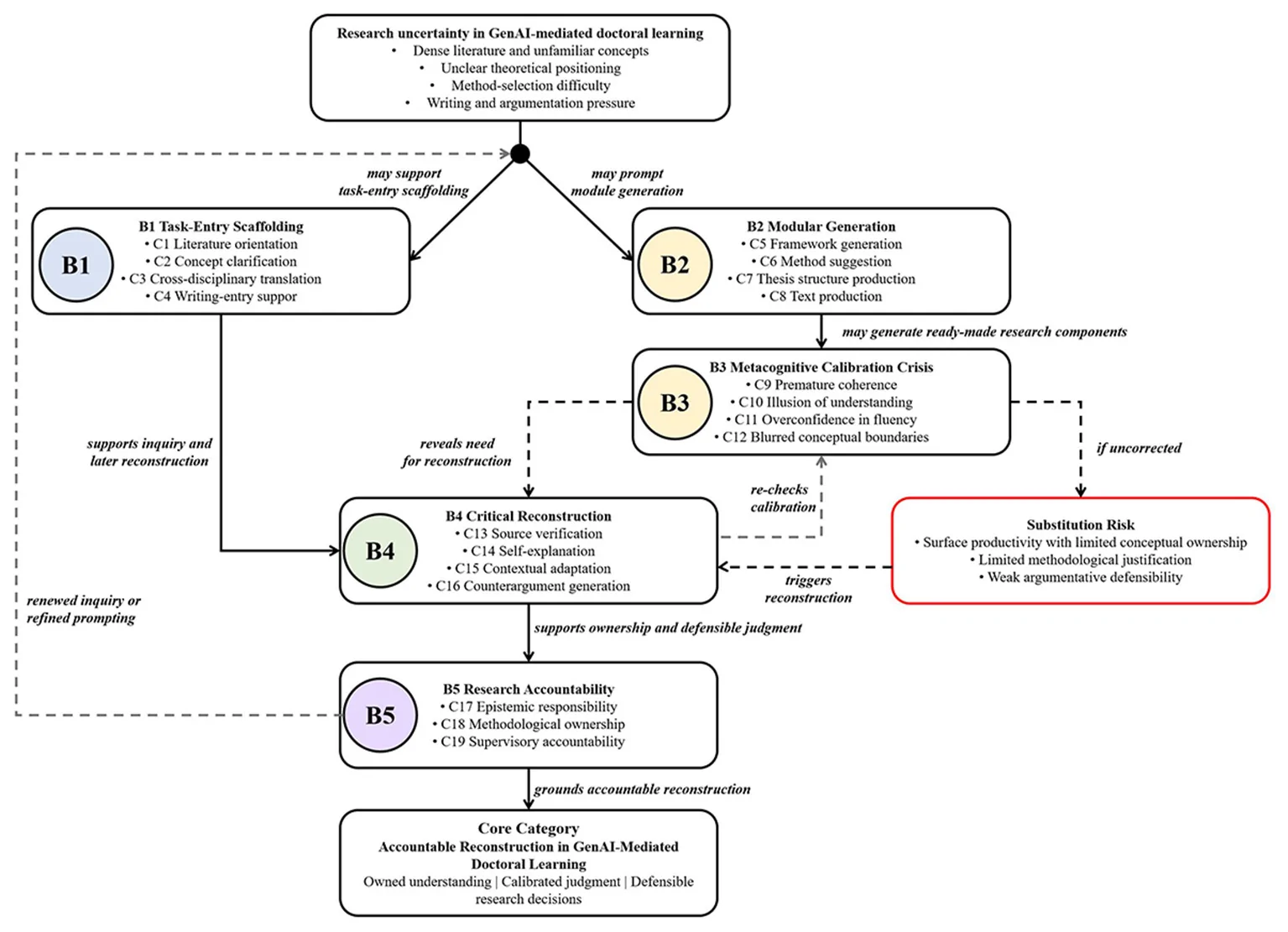
Mechanism model of GenAI-mediated cognitive offloading, metacognitive calibration, and accountable reconstruction in doctoral learning. The model presents analytically related pathways rather than a fixed linear sequence. Task-entry scaffolding and modular generation may co-occur within the same task or participant. Stages may be skipped, repeated, or revisited depending on task demands, disciplinary familiarity, time pressure, supervisory expectations, and learners' metacognitive monitoring. Dashed feedback arrows indicate iterative movement, including re-checking calibration after critical reconstruction and renewed inquiry or refined prompting after research accountability. The feedback from research accountability indicates a return to task-entry scaffolding and/or modular generation rather than a one-way stage sequence. The substitution-risk box indicates a risk pathway rather than an additional grounded-theory main category.

Selective coding integrated the five main categories into a mechanism model of GenAI-mediated doctoral learning as shown in Figure 2. The model begins with research uncertainty. Doctoral students often turned to GenAI when they faced dense literature, unfamiliar concepts, methodological ambiguity, writing pressure, or uncertainty about research structure.

This uncertainty could lead to two analytical pathways. In the first pathway, GenAI supported task-entry scaffolding. Students used AI to orient reading, clarify concepts, translate across disciplinary boundaries, or improve self-drafted writing. Here, cognitive offloading reduced initial difficulty and preserved learner-controlled activities such as source checking and reconstruction. Students returned to sources, checked claims, and reconstructed ideas.

In the second pathway, GenAI produced modular outputs, such as frameworks, methods, structures, hypotheses, or text. These outputs could be useful as provisional resources, and they also created substitution risk when they appeared more coherent than the student's own understanding. Modular generation could therefore produce a metacognitive calibration crisis. Students might mistake fluent AI output for comprehension, methodological readiness, or theoretical justification.

The two pathways are not mutually exclusive. Task-entry scaffolding and modular generation may co-occur within the same participant or even within the same research task. For example, a student may first use AI to clarify a concept, then ask it to generate a framework, and later return to source verification and self-explanation. The model therefore describes a process mechanism rather than a fixed linear sequence. Stages may be skipped, repeated, or reversed depending on task complexity, disciplinary familiarity, time pressure, supervisory expectations, and the learner's metacognitive monitoring. The dashed feedback arrows in Figure 2 further indicate that reconstruction and accountability may reopen earlier parts of the process. Critical reconstruction may lead learners back to calibration checking, whereas research accountability may generate renewed inquiry or refined prompting, returning learners to task-entry scaffolding and/or modular generation.

Substitution risk triggered the need for critical reconstruction. Through source verification, self-explanation, contextual adaptation, and counterargument generation, students addressed the gap between AI-generated output and accountable judgment. Critical reconstruction supported ownership and led to accountable reconstruction, which was expressed as owned understanding, calibrated judgment, and defensible research decisions. In this way, doctoral research agency became visible when students transformed AI-supported outputs into decisions that they could justify, defend, and own.

### 3.8 Cross-case pattern space of GenAI-mediated offloading and reconstruction

**Figure 3:**
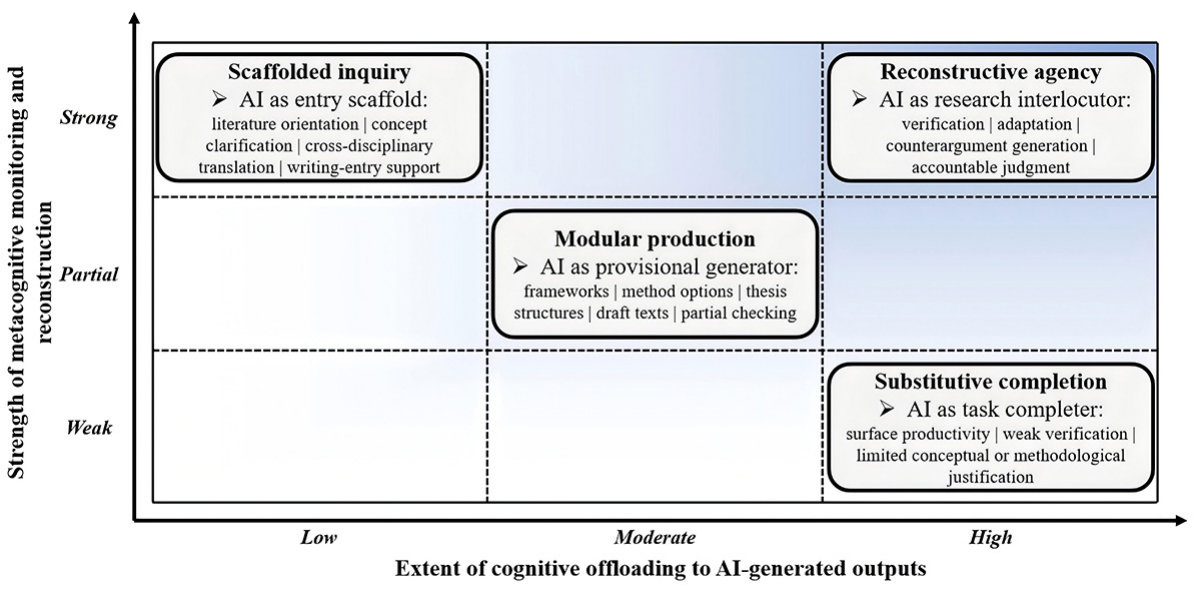
Cross-case pattern space of GenAI-mediated cognitive offloading and metacognitive reconstruction. The four patterns are interpretive configurations of AI-mediated research episodes and recurring task sequences rather than mutually exclusive participant groups. The horizontal axis represents the extent of cognitive offloading to AI-generated outputs, and the vertical axis represents the strength of metacognitive monitoring and reconstruction. The positions indicate analytic tendencies rather than quantitative scores or prevalence estimates.

Cross-case analysis generated four recurring patterns of GenAI-mediated doctoral learning: scaffolded inquiry, modular production, substitutive completion, and reconstructive agency. These patterns describe configurations of AI use across cases rather than additional grounded-theory categories at the same level as the five main categories. The five main categories explain the process mechanism of GenAI-mediated doctoral learning, whereas the four cross-case patterns describe how different degrees of cognitive offloading and metacognitive reconstruction were combined in participants' practices. In this sense, reconstructive agency refers to a broader cross-case configuration in which critical reconstruction practices were combined with sustained learner ownership and research accountability. Table 3 summarizes the analytical distinctions among the four patterns, and Figure 3 positions them within an interpretive pattern space defined by the extent of cognitive offloading and the strength of metacognitive monitoring and reconstruction.

**Table 3:** Cross-case patterns of GenAI-mediated offloading and reconstruction.

Pattern	Configuration of GenAI use	Learner activity	Implication for learner-controlled engagement
Scaffolded inquiry	GenAI is used to enter difficult knowledge domains and clarify unfamiliar research materials.	Students use AI-generated outputs as entry resources, then return to original texts, disciplinary concepts, or research materials.	AI supports manageable task entry while preserving learner-controlled reading, interpretation, and verification.
Modular production	GenAI is used to generate visible components of research work, such as outlines, frameworks, hypotheses, methods, or draft paragraphs.	Students examine the conceptual relations, methodological assumptions, and argumentative logic embedded in AI-generated materials.	AI provides coherent structures for further work, while the extent of learner ownership depends on subsequent examination and reconstruction.
Substitutive completion	GenAI is used to complete research tasks with limited verification or reconstruction.	Students may present polished structures or texts while struggling to explain the reasoning behind claims, methods, or conceptual links.	Generated coherence creates a risk of surface productivity with limited ownership of underlying reasoning.
Reconstructive agency	GenAI is used actively within an iterative process of inquiry, verification, adaptation, and judgment.	Students ask for counterarguments, check references, compare methods, rewrite explanations, and document accepted, modified, or rejected suggestions.	AI-supported outputs are transformed into owned understanding, calibrated judgment, and defensible research decisions.

Together, Table 3 and Figure 3 show how patterns of learner-controlled engagement differed according to the extent to which students retained, monitored, and reconstructed the intellectual work supported by GenAI. Scaffolded inquiry appeared when students used GenAI to enter difficult knowledge domains while maintaining control over reading, interpretation, and verification. Modular production appeared when students used GenAI to generate visible components of research work and then partially examined the conceptual relations, methodological assumptions, or argumentative logic embedded in the generated materials. Substitutive completion appeared when students used GenAI to complete research tasks with limited verification or reconstruction. Reconstructive agency appeared when students used GenAI actively while maintaining strong metacognitive monitoring and research ownership.

Movement across these patterns was shaped by task type, disciplinary familiarity, research stage, time pressure, publication demands, language needs, and supervisory expectations. Humanities and social-science participants more often described conceptual explanation, theoretical framing, source tracing, writing stance, and construct-boundary problems. STEM and applied-science participants more often emphasized method fit, data conditions, technical assumptions, and experimental feasibility. Interdisciplinary participants more often described cross-field translation and the difficulty of judging AI-generated explanations across disciplinary boundaries. These disciplinary differences did not produce separate models, but they shaped where miscalibration was most likely to appear and what forms of reconstruction participants used. These disciplinary comparisons are exploratory and are intended to identify variation in the expression of the mechanism rather than to establish systematic between-discipline differences.

For example, P04 used AI frequently for coding and technical explanation while repeatedly checking AI suggestions against project constraints. P20 used AI less frequently but still struggled to justify method choices when relying mainly on templates and previous theses. These cases indicate that learner ownership differed according to the reconstruction accompanying AI use. The decisive issue was the extent to which AI-supported outputs were transformed into defensible judgment.

## 4 Discussion

This study examined how doctoral students use GenAI in research-related learning and how AI-generated outputs become scaffolds for inquiry or substitutes for learner-controlled reasoning. The findings indicate that learner-controlled engagement differed according to where cognitive and metacognitive work was located. Stronger evidence of learner ownership appeared when AI reduced entry difficulty while students retained responsibility for reasoning, verification, and reconstruction. Risks to ownership appeared when generated coherence was accepted before students had reconstructed the underlying conceptual, methodological, or argumentative logic. These findings concern process-level patterns of engagement rather than demonstrated improvements in learning outcomes.

### 4.1 GenAI-mediated cognitive engagement in high-complexity learning

GenAI-mediated engagement in doctoral learning should be interpreted through learners' ownership of reasoning, not only through the visibility or fluency of final outputs. Participants often used GenAI to produce outlines, explanations, frameworks, method options, or revised paragraphs that appeared coherent and academically usable. These visible products did not necessarily indicate that learners had developed ownership of the reasoning behind them. This distinction extends recent work showing that AI assistance can support learning, writing, feedback, and knowledge access while also reshaping student agency and self-regulated learning processes (Darvishi et al., 2024; Fan et al., 2025; Xu et al., 2025).

In doctoral learning, cognitive engagement became visible when students could reconstruct and defend the reasoning behind a coherent outline, framework, method plan, or paragraph. The issue is therefore not simply whether AI improves the immediate quality or efficiency of an academic product, but whether students can verify, adapt, explain, and defend the intellectual work that the product appears to contain. This distinction is especially important in doctoral research because claims, methods, and arguments must withstand supervisory and disciplinary scrutiny. GenAI-supported work became educationally meaningful when it moved students further into inquiry, source checking, and accountable judgment rather than merely producing a polished external result.

### 4.2 Productive and substitutive cognitive offloading

The distinction between productive and substitutive offloading emerged from how participants redistributed research work between themselves and GenAI. In task-entry scaffolding, students used GenAI to orient themselves to dense literature, clarify concepts, translate across disciplinary boundaries, and improve self-drafted writing. In these cases, AI lowered the entry threshold while students continued to read, verify, and reconstruct ideas. Such use is consistent with recent evidence that GenAI tools can support academic task performance when learners remain engaged in regulation, shared metacognition, and reflective use of AI-generated outputs (Iqbal et al., 2025; Xu et al., 2025).

The same process became more problematic when offloading moved into core research reasoning. Modular generation gave students ready-made frameworks, method suggestions, thesis structures, and polished text. These outputs could serve as useful provisional resources, but they also created the possibility that conceptual, methodological, or argumentative work would be relocated away from the learner. The doctoral cases add an important qualification to current discussions of AI assistance and learner agency: the risk does not lie in offloading itself, but in offloading the kinds of intellectual work through which doctoral students learn to become researchers (Darvishi et al., 2024).

This distinction also clarifies the relationship between cognitive offloading and cognitive outsourcing. In this study, cognitive offloading refers to the redistribution of cognitive work to an external tool. It becomes educationally meaningful in the present cases when learners use the tool to support deeper inquiry. Cognitive outsourcing refers more broadly to delegating task responsibility to another agent. The problematic cases in this study were therefore offloading episodes that approached substitution because students no longer reconstructed or defended the reasoning embedded in AI-generated outputs. The same visible act of using GenAI may represent very different learning processes depending on whether the learner remains responsible for verification, interpretation, and final judgment.

### 4.3 Premature coherence and metacognitive miscalibration

One of the clearest risks identified in the study was premature coherence. Participants repeatedly described situations in which AI-generated explanations, frameworks, or method suggestions appeared organized and convincing before they had developed corresponding understanding. Such coherence could create a misleading sense that a conceptual problem had been clarified, a theoretical relation had been justified, or a method had been selected appropriately.

Premature coherence extends recent concerns about metacognitive laziness in GenAI-supported learning (Fan et al., 2025), but it identifies a more specific calibration problem. Metacognitive laziness concerns reduced willingness or effort to engage in metacognitive regulation. Metacognitive calibration crisis concerns the accuracy of learners' judgments about whether AI-generated coherence corresponds to their own understanding. Reduced monitoring effort may contribute to calibration crisis, but calibration crisis may also arise when learners monitor actively yet rely on misleading cues such as fluency, apparent completeness, or generic coherence. The problem observed here was therefore a specific form of metacognitive miscalibration: students sometimes mistook recognition of AI-generated fluency for ownership of research reasoning. Several problematic outputs were plausible enough to reduce further inquiry, which made them difficult to detect as risks. In this sense, fluent GenAI output can be educationally consequential even when factual errors are not immediately visible.

The characteristics of GenAI outputs intensified this calibration problem. Fluency made outputs feel professionally acceptable. Generic coherence made frameworks and outlines appear complete. Source opacity made it difficult to know whether a claim was grounded in literature. Context-insensitive method advice made technically plausible suggestions appear appropriate before data conditions and assumptions had been examined. These output characteristics interacted with learners' time pressure, disciplinary unfamiliarity, writing anxiety, and supervisory expectations. The resulting risk was a weakened process of monitoring, checking, and reconstruction.

The doctoral context makes this miscalibration particularly important. In course-based learning, overconfidence may appear as a mistaken belief that one has understood material before an assignment or assessment. In doctoral learning, miscalibration may enter a theoretical framework, a method choice, a literature gap, or an argumentative structure. Xu et al. (2025) show that metacognitive support can improve self-regulated learning and reduce cognitive load in GenAI-supported learning. The present study specifies the kind of calibration work needed in doctoral research practice: students need opportunities to test whether generated coherence can be translated into explanation without AI wording, source-based justification, method-data fit, and defensible argumentation.

### 4.4 Critical reconstruction as self-regulated GenAI learning

Critical reconstruction was the main process through which students repaired the gap between AI-generated output and owned understanding. It included source verification, self-explanation, contextual adaptation, and counterargument generation. These practices correspond closely to self-regulated learning processes, but they appeared here in research-specific forms. Source verification functioned as epistemic monitoring. Self-explanation functioned as calibration checking. Contextual adaptation functioned as strategic control. Counterargument generation functioned as reflective evaluation.

Critical reconstruction gives concrete content to recent discussions of GenAI literacy and metacognitive support. In higher education, GenAI literacy increasingly concerns not only technical familiarity with AI systems, but also the ability to critically evaluate, ethically incorporate, and appropriately act on AI-generated outputs (Jin et al., 2025). In doctoral research learning, this general ability took a more specific form: students needed to reconstruct AI-supported outputs into claims, methods, and arguments they could defend. Similarly, metacognitive support in GenAI environments should not be limited to general reminders to monitor understanding. In the doctoral cases examined here, effective support required concrete reconstruction practices, including tracing AI-generated claims to original sources, explaining concepts without AI wording, checking whether suggested methods fit data conditions, adapting AI-generated structures to disciplinary purposes, and using counterarguments to test the strength of one's own claims (Xu et al., 2025).

The results also complicate simple accounts of AI dependency. Dependency was not equivalent to frequent use, and agency was not guaranteed by infrequent use. Some participants used AI intensively while maintaining strong control because they treated AI as an interlocutor for testing, comparison, and revision. Others used AI less often but still showed limited ownership when they accepted templates or structures without examining their assumptions. Critical reconstruction therefore provides a more precise criterion for evaluating GenAI-supported learning: the learner must be able to show how AI output was checked, revised, situated, and incorporated into their own judgment.

### 4.5 Accountable reconstruction and learner ownership in doctoral research learning

Research accountability emerged when students could explain why an AI suggestion was accepted, modified, or rejected, and how final claims, methods, or arguments became their own responsibility. Research accountability connects closely with recent doctoral education discussions emphasizing that doctoral education concerns both knowledge products and the formation of the doctoral knower (McKenna, 2025). GenAI can produce outputs that resemble research expertise, but doctoral learning depends on whether students can transform those outputs into accountable research decisions.

Accountable reconstruction differs from general self-regulated learning because it focuses on the point at which AI-supported materials become claims, methods, or arguments that doctoral students are prepared to justify under supervisory and disciplinary scrutiny. It also differs from general GenAI literacy because it concerns the transformation of AI-supported outputs into defensible scholarly judgment within a research-learning process. The theoretical boundary between accountable reconstruction and related constructs is further summarized in Supplementary Table S7. The theoretical contribution of accountable reconstruction lies not in treating source verification, self-explanation, contextual adaptation, or counterargument generation as individually novel practices, but in specifying how these practices operate together to convert AI-generated coherence into defensible scholarly judgment under disciplinary and supervisory accountability.

Recent studies of doctoral AI use, doctoral writing, and AI-assisted supervision help clarify why this boundary matters. Doctoral students already use AI tools across research-related tasks while encountering concerns about reliability, creativity, and responsible use (Akbar, 2025). AI use in doctoral writing involves meaning negotiation, dissertation support, and authorial stance construction rather than simple text production (Parker et al., 2025; Hoomanfard and Shamsi, 2025; Zhao, 2025). These studies indicate that doctoral writing is not merely a language-support task; it is a site where scholarly voice, methodological accountability, and disciplinary membership are formed. The mechanism developed here adds that GenAI-supported doctoral writing and research planning should be evaluated by whether AI-supported materials are reconstructed into claims, methods, and arguments that the doctoral student can explain and defend.

Supervisory accountability was important because it made AI-supported reasoning visible. When supervisors asked students to mark AI-influenced parts and explain why suggestions were accepted or rejected, students were pushed to connect AI outputs with source evidence, methodological assumptions, and argumentative responsibility. This resonates with recent doctoral education research showing that GenAI can reshape supervisory and peer dynamics, create tensions between productivity-oriented research practices and traditional academic norms, and require clearer renegotiation of roles, responsibilities, and communication practices in doctoral training (Lai et al., 2025). It is also consistent with work arguing that AI-assisted doctoral guidance should be understood as a reconfiguration of supervision rather than a replacement for supervisory judgment (Boyd and Harding, 2025; Iatrellis et al., 2025).

Accountable reconstruction also reframes the relationship between academic integrity and learner ownership. Disclosure makes the use of GenAI visible, but disclosure alone does not demonstrate that students understand or own the resulting work. Students may accurately report their use of GenAI while remaining unable to explain the conceptual relations, methodological assumptions, or argumentative logic contained in an AI-supported output. In the episodes examined here, learner-controlled engagement was more evident when students verified evidence, reconstructed reasoning, adapted suggestions to context, and remained accountable for final decisions. Accountable reconstruction therefore provides a process-level account of how AI-supported materials became integrated into students' own scholarly judgment as owned understanding, calibrated judgment, and defensible research decisions. This interpretation concerns process-level indicators of learner ownership and accountable engagement rather than demonstrated improvements in learning performance or research-product quality.

Although the empirical context is doctoral research learning, the mechanism may be analytically relevant to other high-complexity learning tasks in which learners must evaluate, adapt, and defend AI-supported outputs. Such transferability should be understood analytically because the present study develops a qualitative mechanism model grounded in a specific research-learning context.

### 4.6 Practical implications

The findings suggest three implications for the design of GenAI-supported learning in high-complexity academic contexts.

For supervision, doctoral supervisors can incorporate claim-ownership discussions into regular meetings. Supervisors can ask students to identify where GenAI influenced a framework, method choice, paragraph, or claim; explain why specific suggestions were accepted, modified, or rejected; and defend how the final version is connected to literature, data conditions, and disciplinary expectations. This turns AI use from a hidden production aid into a visible object of scholarly reasoning. Recent work on doctoral GenAI use similarly suggests that AI-assisted supervision requires clearer role negotiation among students, supervisors, peers, and AI tools rather than simple permission, prohibition, or replacement of supervisory judgment (Lai et al., 2025; Iatrellis et al., 2025).

For assessment and milestone review, doctoral programs can require AI-output annotation and reconstruction logs. Such logs do not need to reproduce full private prompt histories. They can ask students to document the purpose of AI use, the type of output received, the verification steps taken, the changes made, and the final ownership judgment. These records can help assess learner ownership and accountable engagement by showing whether students verified, adapted, explained, and defended AI-supported work. This implication is consistent with recent studies on GenAI-supported feedback and assessment, which suggest that AI can scaffold higher-level feedback and revision, but that learners still need to evaluate and act on AI-generated input rather than receive it passively (Noroozi et al., 2025).

For instruction, research-methods and academic-writing courses can teach calibration-oriented AI practices. Students can be asked to reconstruct AI explanations without AI wording, trace AI-generated claims to original sources, compare AI method suggestions with data assumptions, and generate counterarguments to AI-supported arguments. These practices can help students move from using GenAI as a production shortcut to using it as a reflective tool for inquiry and judgment. In doctoral research learning, the goal is not only to develop general GenAI literacy, but to cultivate the capacity to transform AI-supported outputs into evidence-based, methodologically appropriate, and defensible scholarly decisions.

### 4.7 Limitations and future research

Several boundaries shape the interpretation of this study. First, the participants were doctoral students from Chinese research universities, where supervisory relationships, publication pressure, English academic writing demands and institutional norms may shape GenAI use in distinctive ways. The model should therefore be interpreted as analytically transferable rather than statistically generalizable. Its most direct transferability is to high-complexity learning contexts where learners must evaluate, adapt, and defend AI-supported outputs.

Second, AI-use artifacts and stimulated-recall participation were voluntary. Students willing to share such records may have been more reflective, confident, AI-literate or normatively careful than those who declined. This may have led to overrepresentation of participants who were already able to articulate AI-use decisions. The study addressed this risk through negative-case analysis, including comparison between participants who used AI frequently while maintaining strong reconstruction practices and participants who used AI less frequently while showing limited conceptual or methodological ownership. Future studies could recruit less confident or less AI-literate users more deliberately and examine unsuccessful, hidden, or abandoned AI-use episodes in greater depth.

Third, stimulated recall provided richer process data than ordinary interviews, while still relying on retrospective interpretation. Fourth, although the study reports participant-level tool and model/version information, it did not experimentally compare the technical performance of different AI systems, model configurations, prompt settings, subscription tiers, or backend model-selection mechanisms. The model/version information was used as contextual information for interpreting participants' GenAI-mediated learning episodes. Different tools and model families may generate outputs with different levels of fluency, source opacity, factual reliability, and context sensitivity, which may affect metacognitive calibration. Future research could combine interview data with systematic prompt-history collection, model-version logs, and tool-comparison designs to examine how specific model characteristics shape cognitive offloading, calibration crisis, and accountable reconstruction. Fifth, although supervisory accountability emerged as an important category, supervisors themselves were not interviewed. Sixth, the qualitative design did not include direct measures of learning performance, knowledge retention, transfer, or the quality of final research products, nor did it include a comparison group or independent outcome ratings. Accordingly, the findings concern process-level indicators of learner ownership, metacognitive monitoring, verification, and reconstruction rather than demonstrated improvements in learning outcomes.

Because the study develops a qualitative mechanism model, future intervention, longitudinal, and mixed-methods studies can operationalize productive offloading, premature coherence, metacognitive calibration crisis, critical reconstruction and accountable reconstruction to test their associations with learning performance, knowledge retention, transfer, and research-product quality over time. Future research could also use writing-version analysis, supervisor-student dyadic data, experimental comparisons, and cross-national samples to examine how GenAI-mediated offloading and accountable reconstruction develop across doctoral trajectories.

## 5 Conclusion

This study developed a grounded mechanism model comprising task-entry scaffolding, modular generation, metacognitive calibration crisis, critical reconstruction, and research accountability. More learner-controlled engagement was observed when GenAI helped doctoral students enter difficult tasks, compare possible directions, and refine their own thinking while preserving responsibility for verification, reasoning, and reconstruction. Risks to learner ownership appeared when generated coherence was accepted before conceptual, methodological, or argumentative ownership had been formed. The central educational challenge is therefore to support accountable reconstruction by helping learners transform AI-supported outputs into owned understanding, calibrated judgment, and defensible research decisions. GenAI-supported doctoral learning should be examined through the reconstruction practices learners use to verify, adapt, explain, and defend AI-supported knowledge work.

## Data Availability

The original contributions presented in the study are included in the article/Supplementary material, further inquiries can be directed to the corresponding author.
