# Supplementary material for: Cognitive offloading and metacognitive calibration in generative AI-mediated doctoral learning: a grounded theory study of accountable reconstruction
Source: Front Psychol. 2026 Aug 31;17:1920947. doi: 10.3389/fpsyg.2026.1920947 (PMC13572693; doi:10.3389/fpsyg.2026.1920947)
Supplement: Supplementary file 1 [file Table_1.docx]

Supplementary Material

# 1 Supplementary Tables

**Supplementary Table S1.** AI-use profile of formal participants.

| **Participant** | **Discipline group** | **Doctoral year** | **Primary AI-supported research tasks** | **Main GenAI tools reported** | **Model/version information** | **Artifacts provided** | **Stimulated recall** |
| --- | --- | --- | --- | --- | --- | --- | --- |
| P01 | Humanities and social sciences | Year 1 | Literature summarization; concept orientation | ChatGPT; Google Gemini | ChatGPT GPT-5; Gemini 2.5 Flash | No | No |
| P02 | STEM and applied sciences | Year 3 | Method comparison; technical explanation | ChatGPT; DeepSeek | ChatGPT GPT-5; DeepSeek-R1 | No | No |
| P03 | Humanities and social sciences | Year 1 | Literature orientation; theory explanation | ChatGPT; Google Gemini | ChatGPT GPT-5; Gemini 2.5 Pro | Yes | Yes |
| P04 | STEM and applied sciences | Year 4 | Coding support; technical explanation | ChatGPT; GitHub Copilot; Claude | ChatGPT GPT-5; GitHub Copilot Chat in IDE extension/plugin environments; Claude Sonnet 4.5 | No | No |
| P05 | Humanities and social sciences | Year 2 | Framework generation; hypothesis development | ChatGPT; DeepSeek; Google Gemini | ChatGPT GPT-5; DeepSeek-R1; Gemini 2.5 Pro | Yes | Yes |
| P06 | Humanities and social sciences | Year 3 | Construct clarification; scale interpretation | ChatGPT; Google Gemini | ChatGPT GPT-5; Gemini 2.5 Flash | Yes | Yes |
| P07 | Humanities and social sciences | Year 2 | Academic writing revision; source checking | ChatGPT; DeepSeek | ChatGPT GPT-5; DeepSeek-V3 | Yes | Yes |
| P08 | Humanities and social sciences | Year 1 | Concept explanation; theoretical reading support | ChatGPT; Google Gemini | ChatGPT GPT-5; Gemini 2.5 Flash | No | No |
| P09 | Humanities and social sciences | Year 4 | Thesis structure production; argument organization | ChatGPT; DeepSeek | ChatGPT GPT-5; DeepSeek-V3 | Yes | Yes |
| P10 | STEM and applied sciences | Year 3 | Cross-disciplinary explanation; mechanism comparison | ChatGPT; DeepSeek; Claude | ChatGPT GPT-5; DeepSeek-R1; Claude Sonnet 4.5 | Yes | Yes |
| P11 | Humanities and social sciences | Year 2 | Counterargument generation; argument refinement | ChatGPT; Google Gemini; Claude | ChatGPT GPT-5; Gemini 2.5 Pro; Claude Sonnet 4.5 | Yes | Yes |
| P12 | STEM and applied sciences | Year 3 | Method suggestion; data-analysis planning | ChatGPT; DeepSeek | ChatGPT GPT-5; DeepSeek-R1 | Yes | Yes |
| P13 | Humanities and social sciences | Year 1 | Literature searching; concept clarification | DeepSeek | DeepSeek-R1 | No | No |
| P14 | STEM and applied sciences | Year 2 | AI-output annotation; supervisory discussion | ChatGPT; DeepSeek | ChatGPT GPT-5; DeepSeek-R1 | Yes | Yes |
| P15 | Humanities and social sciences | Year 3 | Reading support; preliminary writing support | ChatGPT; Google Gemini; Wenxin Yiyan/ERNIE Bot | ChatGPT GPT-5; Gemini 2.5 Flash; ERNIE 4.5 | No | No |
| P16 | STEM and applied sciences | Year 3 | Method fit checking; assumption clarification | ChatGPT; DeepSeek | ChatGPT GPT-5; DeepSeek-R1 | Yes | Yes |
| P17 | Humanities and social sciences | Year 2 | Literature mapping; writing-entry support | ChatGPT; Doubao | ChatGPT GPT-5; Doubao-1.5-Pro | No | No |
| P18 | Humanities and social sciences | Year 2 | Claim verification; source tracing | ChatGPT; DeepSeek; Google Gemini | ChatGPT GPT-5; DeepSeek-R1; Gemini 2.5 Pro | Yes | Yes |
| P19 | STEM and applied sciences | Year 4 | Technical troubleshooting; model explanation | Wenxin Yiyan/ERNIE Bot; Doubao | ERNIE 4.5; Doubao-1.5-Pro | No | No |
| P20 | Humanities and social sciences | Year 1 | Template comparison; proposal orientation | ChatGPT; DeepSeek; Claude | ChatGPT GPT-5; DeepSeek-R1; Claude Sonnet 4.5 | No | No |
| P21 | STEM and applied sciences | Year 3 | Technical explanation; experimental planning | ChatGPT; GitHub Copilot; DeepSeek | ChatGPT GPT-5; GitHub Copilot Chat in IDE extension/plugin environments; DeepSeek-V3 | No | No |
| P22 | Humanities and social sciences | Year 2 | AI-supported writing revision; prompt-response comparison | ChatGPT; Doubao | ChatGPT GPT-5; Doubao-1.5-Pro | Yes | Yes |
| P23 | Interdisciplinary fields | Year 2 | Cross-field concept translation; literature orientation | DeepSeek; GitHub Copilot | DeepSeek-R1; GitHub Copilot Chat in IDE extension/plugin environments | No | No |
| P24 | Humanities and social sciences | Year 2 | Concept clarification; research-question refinement | Wenxin Yiyan/ERNIE Bot | ERNIE 4.5 | No | No |
| P25 | STEM and applied sciences | Year 2 | Method comparison; coding support | ChatGPT; DeepSeek; Claude | ChatGPT GPT-5; DeepSeek-V3; Claude Sonnet 4.5 | No | No |
| P26 | Interdisciplinary fields | Year 3 | Framework comparison; interdisciplinary explanation | ChatGPT; Wenxin Yiyan/ERNIE Bot | ChatGPT GPT-5; ERNIE 4.5 | No | No |
| P27 | STEM and applied sciences | Year 4 | Technical writing; method description polishing | Wenxin Yiyan/ERNIE Bot | ERNIE 4.5 | No | No |
| P28 | Interdisciplinary fields | Year 4 | Literature integration; academic writing support | ChatGPT; Google Gemini; Wenxin Yiyan/ERNIE Bot; Doubao | ChatGPT GPT-5; Gemini 2.5 Pro; ERNIE 4.5; Doubao-1.5-Pro | No | No |

*Note.* Tool use was recorded as multiple-response information because several participants used more than one GenAI tool across different research tasks. “Tool” refers to the user-facing product, platform, or application through which participants interacted with GenAI. “Model/version information” refers to model labels, product-family labels, or application-version information reported by participants or visible in AI-use artifacts. GitHub Copilot was treated as an AI coding assistant used through IDE extension/plugin environments rather than as a general-purpose conversational chatbot. Tool and model/version information was used to contextualize AI-mediated learning episodes and was not used to compare model-level performance.

**Supplementary Table S2.** Interview guide and stimulated-recall protocol.

| **Component** | **Section or step** | **Interview focus** | **Guiding questions or prompts** |
| --- | --- | --- | --- |
| Semi-structured interview | Doctoral research background | Research stage, disciplinary context, and current doctoral tasks | Please briefly describe your doctoral research topic and current stage. What kinds of research tasks are most difficult for you at this stage? |
|  | History of GenAI use | Entry into GenAI use and general usage habits | When did you begin using GenAI for academic or research-related tasks? In what situations do you usually use GenAI during doctoral research? |
|  | GenAI-supported research tasks | Specific uses in research learning | What research-related tasks do you use GenAI for? For example, literature orientation, concept clarification, method comparison, coding support, framework generation, or academic writing. |
|  | Entering difficult research tasks | Use of GenAI to begin complex or unfamiliar research tasks | Can you describe a situation in which GenAI helped you begin a difficult research task? What did the AI output help you notice, understand, or organize? |
|  | Generating research components | Use of GenAI to produce visible parts of research work | Have you used GenAI to produce an outline, theoretical framework, hypothesis, method suggestion, or paragraph? How did you evaluate whether the output was useful? |
|  | Judging understanding and task completion | Learner judgments of comprehension, progress, and readiness | Have you ever felt that an AI-generated answer made you think you understood something, but later found that you could not explain it clearly? What happened? |
|  | Checking, revising, and adapting AI outputs | Practices for verifying, modifying, or challenging AI-generated outputs | How do you check AI-generated claims, concepts, references, methods, or arguments? What kinds of AI suggestions do you usually accept, revise, or reject? |
|  | Defending AI-supported research decisions | Ownership of AI-influenced claims, methods, writing, and arguments | When AI influences your research writing, method choice, framework, or argument, how do you decide whether the final version is something you can defend as your own research judgment? |
|  | Supervisory and institutional context | Guidance, norms, and accountability expectations | Has your supervisor or institution provided any guidance on the use of GenAI? How does this guidance affect your use of AI in research work? |
|  | Tool and model context | GenAI product and model/version context | Which GenAI tools did you mainly use in this episode? If the model or version label was visible, what was shown or what did you select? |
|  | Closing reflection | Overall learning meaning of GenAI use | In your view, when does GenAI help doctoral learning most? When might it become risky for learning or research development? |
| Stimulated recall | Prompt purpose | Purpose of the original AI interaction | What were you trying to achieve when you entered this prompt? |
|  | Tool/model context | Tool and model/version visible in the artifact | Which tool was used in this artifact? Was there a visible model label, product-family label, or selected model? |
|  | Interpretation of AI output | Participant interpretation of the AI response | How did you interpret the AI response at the time? Which parts seemed useful, unclear, or problematic? |
|  | Acceptance and rejection | Decisions about use, revision, or rejection | Which parts did you accept, revise, ignore, or reject? Why? |
|  | Verification process | Checking AI output against sources, data, disciplinary knowledge, or feedback | Did you check the AI output against original literature, data conditions, disciplinary knowledge, supervisor feedback, or other sources? |
|  | Reconstruction process | Transformation of AI output into participant thinking or writing | How did this AI output change your own thinking, writing, framework, method choice, or argument? |
|  | Ownership judgment | Ability to explain and defend the final version | After using or revising the AI output, could you explain and defend the final version in your own words? |
|  | Learning reflection | Retrospective evaluation of learning value | Looking back at this episode, did the AI output help you understand the task better, or did it mainly help you produce a visible result? |

*Note.* The interview protocol was used flexibly rather than as a fixed sequence. Participants were first invited to describe concrete GenAI-supported research episodes in their own terms. The construct-oriented questions listed in the table were used as optional follow-up probes to clarify participants’ experiences after an initial open-ended account had been provided. These prompts guided data elicitation but did not constitute an a priori coding framework. The categories reported in the study were developed through open coding, constant comparison, theoretical sampling, and iterative comparison across interviews, artifacts, and stimulated-recall data.

**Supplementary Table S3.** Coding development and audit trail.

| **Analytic stage** | **Empirical base** | **Analytic operation** | **Main output** | **Audit record** |
| --- | --- | --- | --- | --- |
| Pilot interview refinement | 4 pilot interviews | Tested interview questions, adjusted sequence, and clarified probes about AI-supported research episodes | Revised interview guide | Pilot memos and revised protocol |
| Formal interview data generation | 28 formal interviews | Conducted semi-structured interviews with doctoral students across six Chinese research universities | Verbatim Chinese transcripts | Audio records, transcripts, anonymization log |
| Artifact collection | 42 AI-use artifacts from 12 participants | Collected voluntarily provided prompt-response excerpts, AI-generated outlines, method suggestions, writing traces, and annotations | Artifact corpus | De-identified artifact inventory |
| Stimulated recall | 12 stimulated-recall discussions | Asked participants to reconstruct prompt purpose, output interpretation, acceptance/rejection, verification, and ownership judgment | Episode-level explanations of AI use | Stimulated-recall notes and linked artifact memos |
| Open coding | 22 model-construction interviews and related artifacts | Conducted line-by-line and incident-by-incident coding | 512 meaning units and 63 initial concepts | Open-coding spreadsheet and analytic memos |
| Initial categorization | 63 initial concepts | Compared similar concepts across participants, tasks, and artifact episodes | 19 initial categories | Category comparison table |
| Axial coding | 19 initial categories | Compared conditions, actions/interactions, and consequences | 5 main categories | Axial coding memos |
| Selective coding | 5 main categories | Integrated categories around the core process | Core category: accountable reconstruction in GenAI-mediated doctoral learning | Selective coding memo and model diagram |
| Stimulated-recall integration | 12 stimulated-recall discussions and linked artifacts | Coded interview segments, artifact traces, annotations, and recall explanations together at the episode level | Refined category properties and process relations | Episode-level matrix |
| Constant comparison | Interviews, artifacts, recall data, memos | Compared incidents across participants, disciplines, doctoral years, task types, and AI-use patterns | Strengthened category boundaries | Constant-comparison memo |
| Theoretical sampling | Later recruitment and follow-up questioning | Emphasized cases involving framework generation, method suggestion, writing support, and uncertainty about AI outputs | Elaborated scaffolding, substitution, and reconstruction | Recruitment and follow-up memo |
| Negative-case analysis | Cross-case comparison | Compared frequent AI use with strong reconstruction and less frequent AI use with limited ownership | Clarified that differences in learner ownership were linked to reconstruction practices rather than to AI-use frequency alone | Negative-case memo |
| Two-researcher coding review | 120 double-coded meaning units | Reviewed main-category assignment before discussion and resolved discrepancies through comparison with original materials and analytic memos | Initial raw percentage agreement = 0.86 | Coding-agreement record |
| Translation checking | Representative quotations | Translated quotations after analysis and checked against original Chinese transcripts | Semantically accurate English quotations | Translation-checking notes |
| Saturation testing | 6 reserved formal interviews | Tested whether the five main categories and their relations were sufficiently developed | No new main categories or core relationships emerged | Saturation-testing memo |

**Supplementary Table S4.** Trustworthiness and theoretical saturation checks.

| **Strategy or check** | **Implementation** | **Contribution to trustworthiness** |
| --- | --- | --- |
| Data triangulation | Combined semi-structured interviews, AI-use artifacts, and stimulated-recall discussions | Supported comparison between participants’ accounts, concrete AI-use traces, and retrospective explanations |
| Purposive maximum-variation sampling | Recruited doctoral students across six research universities, disciplines, doctoral years, gender, and AI-use experience | Strengthened analytic transferability |
| Theoretical sampling | Later recruitment and follow-up questioning emphasized participants reporting framework generation, method suggestion, writing support, and uncertainty about AI outputs | Helped elaborate the distinction between scaffolding, substitution, calibration crisis, and reconstruction |
| Constant comparison | Compared incidents across participants, disciplines, doctoral years, task types, and AI-use patterns | Supported category development and theoretical integration |
| Artifact-based comparison | Compared prompt-response records and participant annotations with interview accounts | Clarified how AI-generated outputs entered doctoral research learning practices |
| Stimulated recall | Conducted 12 follow-up discussions with participants who provided usable artifacts | Strengthened episode-level interpretation of how AI outputs were accepted, revised, rejected, verified, or reconstructed |
| Negative-case analysis | Compared participants with frequent AI use and strong agency with participants using AI less frequently but showing limited ownership | Clarified that differences in learner ownership were linked to reconstruction practices rather than to AI-use frequency alone |
| Two-researcher coding review | Two researchers reviewed main-category assignment for 120 double-coded meaning units and resolved discrepancies through discussion | Improved transparency and category consistency; initial raw percentage agreement was 0.86 |
| Analytic memoing | Memos recorded coding decisions, emerging category relations, negative cases, and theoretical integration | Supported an auditable link between empirical materials and theory construction |
| Translation checking | Representative quotations were translated after analysis and checked against the original Chinese transcripts | Preserved semantic accuracy across Chinese data and English reporting |
| Member-checking decision | Formal member checking of final categories was not conducted because participants discussed unpublished doctoral research materials, draft arguments, method choices, or sensitive AI-use records. Stimulated recall, artifact-based comparison, negative-case analysis, two-researcher coding review, and analytic memoing were used to strengthen interpretive credibility | Clarified respondent-validation decision and strengthened transparency of trustworthiness procedures |
| Saturation of task-entry scaffolding | Six reserved interviews repeated examples of literature orientation, concept clarification, writing-entry support, and cross-disciplinary explanation | Category properties confirmed |
| Saturation of modular generation | Six reserved interviews included AI-generated outlines, frameworks, methods, and draft paragraphs | Category properties confirmed |
| Saturation of metacognitive calibration crisis | Six reserved interviews repeated cases of premature coherence, illusion of understanding, overconfidence in fluency, and blurred conceptual boundaries | Category properties confirmed |
| Saturation of critical reconstruction | Six reserved interviews repeated practices of source verification, self-explanation, contextual adaptation, and counterargument generation | Category properties confirmed |
| Saturation of research accountability | Six reserved interviews repeated cases of epistemic responsibility, methodological ownership, and supervisory accountability | Category properties confirmed |
| Core-category saturation | Six reserved interviews supported the relationship among offloading, calibration crisis, reconstruction, and accountability | No new main categories or core relationships emerged |

**Supplementary Table S5.** Representative empirical materials for the 19 initial categories.

| **Main category** | **Initial category** | **Representative empirical material** | **Initial concept** | **Analytical interpretation** |
| --- | --- | --- | --- | --- |
| Task-entry scaffolding | Literature orientation | P03 described using GenAI before reading a dense theoretical article to identify the main debate and key concepts, then returning to the original text for close reading. | AI as reading map | GenAI supported entry into difficult literature while preserving source-based reading. |
|  | Concept clarification | P08 used GenAI to explain unfamiliar philosophical terms before comparing the explanation with assigned readings. | Preliminary concept explanation | GenAI reduced initial conceptual difficulty and helped the participant begin disciplinary reading. |
|  | Cross-disciplinary translation | P10 used GenAI to compare two mechanisms from adjacent fields and then checked the explanation against recent domain papers. | Cross-field language bridge | GenAI supported movement across disciplinary vocabularies while field-specific verification remained necessary. |
|  | Writing-entry support | P07 wrote an initial paragraph independently and then used GenAI to improve clarity and expression. | AI-assisted expression refinement | GenAI supported writing entry when the student’s own meaning preceded AI revision. |
| Modular generation | Framework generation | P05 asked GenAI to generate variables, relationships, and hypotheses for an early-stage research idea. The output looked complete, but the participant could not explain several conceptual links. | Generated theoretical framework | AI generated the external form of theory before the participant had formed theoretical ownership. |
|  | Method suggestion | P12 received several confident method recommendations from GenAI but later found that some did not fit the data structure. | Method options without contextual fit | AI expanded methodological options while leaving assumptions and data fit to the learner. |
|  | Thesis structure production | P09 used GenAI to reorganize a dissertation chapter outline and later found that some transitions were only surface-level headings. | Coherent structure masking gaps | AI-generated organization created a sense of coherence before argumentative continuity was fully established. |
|  | Text production | P22 compared self-written paragraphs with AI-revised versions and noticed that some AI-generated sentences sounded fluent but weakened the intended claim. | Fluent text with diluted meaning | AI-generated text improved readability while requiring careful preservation of argument ownership. |
| Metacognitive calibration crisis | Premature coherence | P05 initially felt that an AI-generated framework was complete because it included variables, relationships, and hypothesis-like statements. | Coherence before justification | Visible coherence created a premature sense of research progress. |
|  | Illusion of understanding | P08 reported that a fluent AI explanation made the concept feel understandable, but later she could only repeat the AI wording. | Recognition mistaken for understanding | The participant recognized plausible wording without being able to reconstruct the argument independently. |
|  | Overconfidence in fluency | P01 described feeling that AI summaries helped cover a field quickly, then realizing that the summary represented only “AI’s version of the field.” | Fluency-based confidence | AI-generated summaries created an emotional sense of control while leaving coverage and interpretation incomplete. |
|  | Blurred conceptual boundaries | P06 found that AI explanations mixed related psychological constructs and blurred distinctions between definition, measurement, and antecedents. | Conceptual boundary loss | AI simplification created clarity at the surface level while weakening construct precision. |
| Critical reconstruction | Source verification | P07 used AI-generated terms to search databases and cited only papers that she had read and verified. | Source tracing | Verification reconnected AI-generated claims to scholarly evidence. |
|  | Self-explanation | P03 closed the AI page and attempted to explain a theory independently to test whether she had understood it. | Explanation without AI wording | Self-explanation functioned as a calibration check for owned understanding. |
|  | Contextual adaptation | P16 compared AI method suggestions with recent field papers, assumptions, and data conditions. | Method fit checking | The learner adapted or rejected AI suggestions according to disciplinary and empirical constraints. |
|  | Counterargument generation | P11 asked AI to criticize an argument and defend the opposite position before revising the argument independently. | AI-supported argument testing | AI became a dialogic resource for reflective evaluation and argument refinement. |
| Research accountability | Epistemic responsibility | P18 stated that an AI-generated claim could enter a paper only after the original source had been read and its relevance could be explained. | Claim ownership | The learner distinguished between possible AI suggestions and defensible research claims. |
|  | Methodological ownership | P02 emphasized that method choices remained the student’s responsibility because AI did not know the equipment, data, and research question in full. | Responsibility for method choice | The learner treated AI as an option generator while retaining responsibility for methodological decisions. |
|  | Supervisory accountability | P14 reported that the supervisor asked students to mark AI-influenced parts and explain accepted or rejected suggestions. | Visible AI-use justification | Supervisory questioning made AI-supported research decisions more explicit and accountable. |

**Supplementary Table S6.** De-identified AI-use artifact episodes.

| **Participant** | **Artifact type** | **Prompt purpose** | **AI-output type** | **Participant annotation or recall explanation** | **Related category** |
| --- | --- | --- | --- | --- | --- |
| P03 | Prompt-response excerpt for theoretical reading | Asked GenAI to outline a theoretical debate before reading the original article | Summary of debate, key concepts, and conceptual tensions | The participant marked the response as a “reading map” and used it to decide where to focus during close reading of the original text. | Literature orientation;task-entry scaffolding |
| P05 | AI-generated conceptual framework | Asked GenAI to generate variables, relationships, and hypotheses for an early research idea | Framework-like model with linked constructs and directional hypotheses | The participant annotated several links as “unclear source” and “needs literature support,” explaining that the model looked complete but could not yet be defended. | Framework generation; premature coherence |
| P06 | AI explanation of psychological constructs | Asked GenAI to compare two related constructs and explain their differences | Simplified conceptual explanation | The participant highlighted places where the AI response blurred construct definition, measurement indicators, and theoretical antecedents. | Concept clarification; blurred conceptual boundaries |
| P07 | AI-assisted writing revision | Provided a self-written paragraph and asked GenAI to improve clarity | Revised academic paragraph | The participant accepted some wording changes but rejected sentences that changed the intended claim or removed source-specific meaning. | Writing-entry support; contextual adaptation |
| P09 | AI-generated thesis outline | Asked GenAI to reorganize the chapter structure of a dissertation section | Hierarchical chapter outline with headings and transitions | The participant later noted that some transitions sounded smooth but did not reflect real argumentative connections. | Thesis structure production; premature coherence |
| P10 | Cross-disciplinary mechanism explanation | Asked GenAI to compare two mechanisms from adjacent scientific fields | Comparative explanation with simplified terminology | The participant marked several statements as too general and checked them against recent domain papers. | Cross-disciplinary translation; contextual adaptation |
| P11 | AI-generated counterarguments | Asked GenAI to criticize a draft argument and defend an alternative interpretation | Counterargument list and alternative reasoning | The participant used the output to identify weak points, then rewrote the argument independently rather than copying the response. | Counterargument generation; critical reconstruction |
| P12 | Method recommendation record | Asked GenAI to suggest suitable analytical models for a planned empirical study | List of possible methods with brief explanations | The participant rejected several suggestions because they did not match the data structure or assumptions of the study. | Method suggestion; methodological ownership; contextual adaptation |
| P14 | AI-output annotation for supervisory discussion | Marked AI-influenced parts of a research draft before supervision | Annotated draft showing accepted and rejected AI suggestions | The participant explained that supervisory questioning required him to justify why each AI suggestion was accepted, modified, or rejected. | Supervisory accountability; research accountability |
| P16 | Method fit-checking artifact | Asked GenAI to explain assumptions behind a possible method | Explanation of assumptions and potential limitations | The participant compared the AI output with recent field papers and data conditions before deciding whether the method was appropriate. | Contextual adaptation; methodological ownership |
| P18 | Source-tracing artifact | Asked GenAI to identify possible sources supporting a claim | Suggested source list and claim explanation | The participant used the output only as a search cue and included the claim only after reading and verifying the original paper. | Source verification; epistemic responsibility |
| P22 | Prompt-response comparison for writing revision | Compared AI-revised paragraph with the participant’s original paragraph | Polished paragraph with improved fluency | The participant accepted expression-level changes but restored original wording where the AI version weakened conceptual precision. | Text production; contextual adaptation |

*Note.* The table reports selected representative artifact-based episodes from the 42 AI-use artifacts voluntarily provided by 12 participants. The episodes are included to illustrate analytic processes and category development rather than to reproduce all prompt histories or disclose participants’ unpublished research materials.

**Supplementary Table S7.** Conceptual comparison between accountable reconstruction and related learning constructs.

| **Construct** | **Theoretical focus** | **Unit of analysis** | **Role of AI** | **Central mechanism** | **Analytical criterion in this study** | **Boundary from accountable reconstruction** |
| --- | --- | --- | --- | --- | --- | --- |
| Cognitive offloading | Redistribution of cognitive work to external resources | Learner-tool interaction | AI functions as an external resource that may reduce cognitive demands or support task management | Redistribution of memory, reasoning, search, explanation, or production work | Offloading was interpreted as productive in this study when it supported further inquiry and did not replace learner reasoning | Accountable reconstruction focuses on how offloaded outputs are verified, adapted, explained, and defended as scholarly judgment |
| Cognitive outsourcing | Descriptive expression for delegating task responsibility to another agent | Task responsibility | AI may be treated as an agent to which task completion is delegated | Responsibility for task completion shifts away from the learner | The expression identifies a risk of responsibility transfer rather than a desired learning process | Accountable reconstruction requires that learners retain responsibility for claims, methods, arguments, and final decisions |
| Cognitive substitution | Replacement of learner reasoning by AI-generated outputs | AI-mediated research episode | AI produces frameworks, methods, structures, or texts that learners may accept without sufficient reconstruction | AI-generated coherence replaces conceptual, methodological, or argumentative work | Substitution risk is reduced when learners reconstruct and defend the reasoning behind AI-supported outputs | Critical reconstruction is the proximal process through which substitution risk is addressed; accountable reconstruction is the broader process through which reconstructed outputs become owned, calibrated, and defensible research decisions |
| Self-regulated learning | Monitoring, control, and strategic adjustment of learning | Learner regulation process | AI becomes an object of monitoring, control, and strategy adjustment | Learners plan, monitor, evaluate, and regulate their engagement with AI outputs | Learners can monitor understanding, adjust strategies, and evaluate progress | Accountable reconstruction specifies how self-regulation operates when AI-supported materials become doctoral claims, methods, or arguments |
| GenAI literacy | Understanding, evaluating, and responsibly using GenAI systems | Learner competence in AI use | AI is a tool whose outputs need to be interpreted, evaluated, and used responsibly | Critical evaluation, ethical use, awareness of limitations, and appropriate action on AI outputs | Learners understand AI affordances and limitations and use AI responsibly | Accountable reconstruction is more specific to doctoral research learning because it concerns defensible scholarly judgment under disciplinary and supervisory accountability |
| Metacognitive laziness | Reduced willingness or effort to engage in metacognitive regulation | Learner effort and regulation | AI may reduce learners’ motivation or effort to monitor and evaluate understanding | Reduced monitoring effort, reduced reflective engagement, or passive acceptance of AI assistance | Risk was lower in episodes where learners actively monitored and regulated AI-supported learning | Accountable reconstruction identifies the practices through which learners repair or avoid miscalibration by verifying, adapting, explaining, and defending AI outputs |
| Metacognitive calibration crisis | Misjudgment of the gap between AI-generated coherence and owned understanding | Learner judgment of understanding | AI-generated fluency, coherence, or completeness may serve as misleading cues | Recognition of fluent output is mistaken for understanding, mastery, or justification | Learners regain calibration when they test whether generated coherence can be translated into source-based justification and independent explanation | Accountable reconstruction describes the broader process through which calibration is restored and AI-supported materials become defensible research decisions |
| Critical reconstruction | Self-regulated practices for working on AI-supported outputs | Reconstruction practice | AI outputs become provisional materials for verification, adaptation, explanation, and challenge | Source verification, self-explanation, contextual adaptation, and counterargument generation | Learners can show how AI output was checked, revised, situated, and incorporated into their own judgment | Critical reconstruction is a main process within accountable reconstruction; accountable reconstruction also includes the final responsibility for claims, methods, and arguments |
| Research accountability | Responsibility for final claims, methods, and arguments | Scholarly judgment | AI may suggest possibilities, but the learner remains accountable for final research decisions | Epistemic responsibility, methodological ownership, and supervisory accountability | Learners can justify, defend, and own final scholarly decisions | Research accountability is the endpoint through which accountable reconstruction becomes visible in doctoral learning |
| Accountable reconstruction | Transformation of AI-supported outputs into owned understanding, calibrated judgment, and defensible research decisions | AI-mediated doctoral research-learning episode | AI provides outputs that may scaffold inquiry, create substitution risk, or trigger reconstruction | Integrated process of offloading, calibration, critical reconstruction, and research accountability | AI-supported materials become claims, methods, or arguments that the doctoral student can explain, verify, adapt, and defend | Study-derived core category; its contribution lies in specifying how established reconstruction practices operate together to convert AI-generated coherence into defensible scholarly judgment under disciplinary and supervisory accountability |
